# Supplementary material for: Content-rich biological network constructed by mining PubMed abstracts
Source: BMC Bioinformatics. 2004 Oct 8;5:147. doi: 10.1186/1471-2105-5-147 (PMC528731; doi:10.1186/1471-2105-5-147)
Supplement: Additional File 5 — The original Chilibot query results of the term "long-term potentiation (LTP)" and 22 other terms, limiting the latest references analyzed to the years 1990, 1995, 2000, and 2004. [file 1471-2105-5-147-S5.bz2 › chilibotAdditionalFile5/ltp1995/html/PI-3K_PKA.html]

 


 **PI-3K** and **PKA** 
  
Found 3 abstracts in PubMed,  **3 abstracts were retrieved and analyzed**.  


---

 Search Google  |
 PDF files only 
|  EDU domain only 

---

- Ann N Y Acad Sci, 1995   **Platelet derived growth factor.
  Distinct signal transduction pathways associated with migration versus proliferation.** .
  Figure 2 summarizes our current interpretation of data concerning signals from the activated PDGF receptor involved in directed migration and proliferation of human arterial SMC.
  Binding of PDGF PDGF BB or PDGF AA causes PDGF receptor dimerization, tyrosine autophosphorylation, and subsequent binding of several molecules containing SH2 domains to the activated receptor.
  Binding and activation of PLC gamma by the PDGF receptor leads to PIP2 hydrolysis, resulting in generation of diacylglycerol DAG and IP3.
  Subsequently, intracellular levels of calcium are elevated as a result of IP3 mediated calcium release from intracellular compartments.
  The decreased levels of PIP2 and increased levels of calcium both favor actin filament disassembly by inducing capping of actin filament barbed ends and actin monomer sequestration.
  A localized, and transient, actin filament disassembly enables the cell to extend filopodia towards PDGF, thereby enabling chemotaxis to take place.
  At a later time and or in a different compartment, actin filament assembly is promoted by PDGF by a mechanism that is not completely understood, but that may involve small GTP binding proteins, such as Rho, and formation of DAG.
  Migration on collagen requires functional alpha 2 beta 1 integrins, which may either constitute a permissive state required for a cell to migrate, or which may be actively involved in intracellular signals leading to migration.
  PDGF induced DNA synthesis and proliferation involves activation of Ras, MAP kinase kinase, and MAP kinase.
  Cross talk between **PKA** signaling and tyrosine kinase receptor signaling results in **PKA** inhibition of the MAP kinase cascade, probably at the level of Raf.
  Activation of PI 3 kinase **[PI-3K]**, or a PI 3 kinase **[PI-3K]** like enzyme, is also likely to contribute to the mitogenic effects of PDGF in these cells Bornfeldt, unpublished observation .
  What determines if a SMC will migrate and or proliferate in response to PDGF? Results are starting to emerge that show regulation of expression of molecules involved in intracellular signaling with different phenotypic states of SMC.
  For example, expression of PLC gamma is very low in intact vascular wall where SMC show a contractile phenotype , and induced when SMC are converted to a synthetic phenotype in culture.
  Proliferation and expression of MAP kinase, but not calcium signaling, appear to be regulated by the extracellular matrix, and the profile of integrin expression is different in SMC in culture compared to SMC in the vascular wall.
  Thus, the relation between expression of signaling molecules involved in migration and signaling molecules involved in proliferation, as well as cross talk between different signal transduction pathways, may determine the net effect of PDGF.

  - Res Commun Mol Pathol Pharmacol, 1995   **Phosphatidylinositol 3 kinase inhibitor, wortmannin, inhibits 5 hydroxytryptamine mediated potentiation of platelet aggregation induced by epinephrine.** .
    The role of phosphatidylinositol 3 kinase PI 3 kinase **[PI-3K]** was investigated in human platelets exposed to varying doses of 5 hydroxytryptamine 5 HT and subthreshold doses of epinephrine.
    The synergistic effect of 5 HT on epinephrine induced aggregation was blocked by a specific inhibitor of PI 3 kinase **[PI-3K]**, wortmannin, in a dose dependent manner.
    However, there was no effect of inhibitors of protein kinase A **PKA** or protein kinase C PKC .
    These studies suggest a role of PI 3 kinase **[PI-3K]** in the 5 HT induced potentiation of epinephrine mediated platelet aggregation.

    - J Cell Physiol, 1995   **Perturbation of the platelet derived growth factor receptor signaling by dibutyryl cAMP in human astrocytoma cells.** .
      It has been shown that cAMP may perturb the polypeptide growth factor induced nuclear events.
      However, the possible interactions of the cAMP protein kinase A cAMP **PKA** and receptor tyrosine kinase pathways in the cytosol have not been fully elucidated.
      In this study, we use human astrocytoma cells as a model to investigate this issue.
      The results show that platelet derived growth factor PDGF induced receptor autophosphorylation in human astrocytoma cells is suppressed by dibutyryl cAMP pretreatment and such suppression is not due to changes in the ligand receptor binding properties.
      Further studies show that PDGF induced tyrosine phosphorylation of phospholipase C gamma 1 PLC gamma 1 and phosphatidylinositol 3 kinase PI 3 kinase **[PI-3K]** are also suppressed in dibutyryl cAMP pretreated cells.
      The suppression of PLC gamma 1 tyrosine phosphorylation was accompanied by a decreased production of water soluble inositol phosphates.
      In contrast, similar treatment with normal human astrocytes potentiates the tyrosine phosphorylation of PLC gamma 1 and PI 3 kinase **[PI-3K]**.
      The results indicate that cAMP can either negatively or positively modulate the PDGF receptor tyrosine kinase activity depending on the cell types examined.
